# Impact on habitual crossfit participant's exercise behavior, health, and well‐being: A cross‐sectional survey of UK COVID‐19 lockdowns

**DOI:** 10.1002/hsr2.1140

**Published:** 2023-02-28

**Authors:** Athalie Redwood‐Brown, Jennifer Wilson, Paul Felton

**Affiliations:** ^1^ School of Science and Technology: Sport and Exercise Sciences Erasmus Darwin 244 Nottingham Trent University Nottingham UK; ^2^ College of Science and Engineering: Sport and Exercise Science University of Derby Derby UK

**Keywords:** COVID‐19, CrossFit™, functional exercise, health, physical activity

## Abstract

**Background and Aims:**

The period between March 2020 and March 2021 saw an unprecedented change to everyday life due to the COVID‐19 pandemic. This included the closure of businesses in the health and fitness sector. Such closures impacted people in several ways; increasing stress, reducing mental well‐being, and decreasing motivation to exercise. The purpose of this study was to evaluate the effect of UK lockdowns on the behavior, motives, and general health & well‐being of CrossFit™ gym members in the United Kingdom.

**Methods:**

A cross‐sectional study was conducted on 757 CrossFit™ participants (height 1.71 ± 0.10 m; weight 76.4 ± 16.1 kg; body mass index [BMI]: 26.1 ± 4.7 kg/m²) using an online survey, which included questions pertaining to COVID‐19, lockdown behaviors, motivation, health, and well‐being. Participants also reported on their training background and exercise habits during lockdown restrictions.

**Results:**

Differences were observed in levels of exercise (*p* = 0.004), motivation to train at home (*p* < 0.001), and the feeling of being more stressed during the second lockdown compared with the first lockdown (*p* = 0.008). It was also highlighted that motivation to exercise was lower and stress levels significantly higher, in the 18–24 and 25–34 age groups compared with older ages groups.

**Conclusion:**

This study found that exercise behavior, motivation, and stress levels were significantly impacted by the second government‐imposed lockdown. It is argued that these factors need to be addressed in planning for future National lockdowns to maintain the health and well‐being of UK residents, especially in younger adults.

## 1 INTRODUCTION

There is an abundance of research linking regular exercise with improved mental and physical health.^1^, ^2^ This relationship is further strengthened by research such as Weinstein et al.,^1^ who investigated the relationship between exercise/physical activity withdrawal and mental health. They found withdrawal from exercise for as little as 2 weeks resulted in symptoms of depression, fatigue, tension, and decreased self‐esteem. Similar feelings were also reported by Antunes and colleagues^3^ who found increases in negative mood following exercise deprivation in exercise addicted subjects.

Over the last 3 years, we have seen several disruptions to everyday life due to government‐imposed lockdowns. One of the major consequences of this has been the reduction in physical activity undertaken, ultimately leading to decreases in mental and physical health, and increases in unhealthy behaviors^4^, ^5^, ^6^, ^7^, ^8^, ^9^ such as poor sleeping patterns/modified sleeping behavior,^6^ poor nutritional choices,^7^ and increased alcohol consumption.^8^ Studies reporting on the health of the general population during the first UK lockdown reported increased sedentary behavior, greater disease risk, and increased negative psychological characteristics.^10^, ^11^, ^12^, ^13^, ^14^, ^15^ These characteristics include posttraumatic stress symptoms, anger, infection fears, and boredom^16^ many of which were considered a consequence of financial struggles, frustration, and inadequate supplies of essential items and services during the lockdown period.^16^

For those who exercise consistently as part of an active lifestyle, gym closures and competition cancellations have been associated with additional stress, anxiety, frustration, and depression, namely related to the removal of social support and the change to normal training/exercise routines.^17^, ^18^, ^19^, ^20^ This reduction in physical activity due to gym closures not only facilitates sedentary behavior but also reduces social interaction which for a lot of individuals impacts mental health and well‐being, particularly as many participate to reduce their symptoms of anxiety and depression.^15^, ^19^ Some preliminary investigations have demonstrated that if exercise behaviors are maintained during lockdown periods, the impact on mental health is limited,^18^ while others have noted a decline in mental health, suggesting that further investigation is needed.^20^ However, it is evident that habitually trained individuals from community focused exercise programs are more likely to maintain exercise habits during nationally imposed lockdowns and report fewer changes to mental health as a result.^18^ Community‐focused programs, such as CrossFit™ are generally associated with high levels of retention and adherence compared with other forms of fitness training,^21^ particularly when the methodology seeks to increases exercise enjoyment; provides challenge and satisfaction; and enhances goal achievement, elements associated with positive mental health.^22^

To understand the impact of restricted exercise access, for population groups such as this, further investigation is required. Specifically, the impact of successive lockdowns on habitual community focused/group exercise and health. Recent investigations by Ocobock and Hejtmanek^20^ support the notion that individuals who participate in CrossFit™ are less likely to be impacted by lockdown, compared with other forms of exercise. However, it is not yet known how multiple lockdown periods may impact participants who are more socially invested in their physical training regimes and thus, could be more vulnerable when normal training regimes are significantly disrupted.^21^ No investigations to date have compared the outcomes of two successive lockdowns, or the long‐term impact on psychological well‐being in habitually trained individuals therefore, this area warrants further exploration.

### 1.1 Aims and objectives

Subsequently, the aims of this study were to investigate the effects of consecutive lockdown periods in a habitually trained population. To inform findings, this study will focus on habitually trained CrossFit™ athletes, where typical levels of retention and adherence are known to be high compared with other forms of fitness training.^18^ Specifically, the study will investigate motivations, exercise behaviors, and general well‐being between the two lockdowns. It is proposed that these findings may help to evidence the importance of gyms and thus their essential status in future lockdowns or prolonged periods of imposed closure.

## 2 MATERIALS AND METHODS

### 2.1 Participants

A total of 757 CrossFit™ participants residing in the United Kingdom were recruited via email invitation sent to 650 UK CrossFit™ affiliate gyms or social media advertisement (Twitter, Facebook, and Instagram) to voluntarily participate in this study. Each participant fully completed an online survey hosted by Survey Monkey which controlled for repeat participants (https://www.surveymonkey.co.uk/) within a 2‐month period (January 8–March 31, 2021). Study details were explained to each participant and informed consent was gathered in accordance with guidelines approved by Nottingham Trent Universities noninvasive Research Ethics Committee. No incentives were offered for participation, nor were there any penalties for not participating (researchers were blind to participation). Responses were removed from analysis if the participant did not reside in the United Kingdom or the survey was incomplete (104 in total).

### 2.2 Survey design

The survey (Appendix 1 available at request from corresponding author) adopted was specifically designed for this study and centered on a self‐reported structure. The questionnaire consisted of five parts. The first part gathered demographic information and consisted of eight questions regarding participant's gender, age, body height, body weight, ethnicity, and training location. The second part of the survey focused on the participants’ training background and consisted of four questions designed using a deductive approach to collect information regarding their training years, the type of gym they train at; the number of minutes exercised per week, and how many CrossFit™ sessions they participate in per week. The third part of the questionnaire focused on participants' behavior, motives, and general health and well‐being during lockdown periods. Participants were asked 12 questions designed using a deductive approach to collate data on whether they felt more stressed, more relaxed, less motivated to train, and exercised less during the second lockdown compared with the first lockdown. Participants were also asked to rank their main motives for participation in CrossFit™, as well as those they had missed most due to the lockdown restrictions. The motives derived using a deductive approach were fitness, mental health, general health, strength, time out, weight, performance, and toning. The fourth part of the survey asked participants about medical conditions and their severity. The final part of the survey asked participants whether they or the people around them had contracted COVID‐19, and the severity of the virus.^17^ In total, there were 5 parts, and a total of 39 questions.

The number of questions for each section was kept to a minimum to reduce response bias associated with boredom and increase validity. For the third section, where participants were asked to respond to statements focused on how the two UK lockdowns had affected their motivations, behaviors, and general health and well‐being, a five‐point Likert‐type scale was adopted to elicit the strength of the participants’ agreement, as follows: 1 = strongly agree; 2 = agree; 3 = neither agree nor disagree; 4 = disagree; 5 = strongly disagree. Each statement was kept as short as possible to improve the validity of the responses. In this section, participants were also asked to rank the order of potential motivations for participation in CrossFit™ by ranking them in order from 1 to 8.

### 2.3 Data processing

All data collected from this study was downloaded and imported into SPSS v.27 (IMB) for processing and statistical analysis where an alpha value of 0.05 was used to determine significance. Data were grouped using three polytomous questions from the survey describing gender, ethnicity, and age. While a further grouping variable was determined by grouping participants based on their body mass index (BMI): underweight (<18.5 kg/m^2^), normal (18.5–24.9 kg/m^2^), overweight (25.0–29.9 kg/m^2^), Obese I (30.0–34.9 kg/m^2^), Obese II (35–39.9 kg/m^2^), Obese III ( > 40 kg/m^2^).

### 2.4 Statistical analysis

To investigate the motives for CrossFit™ participation the responses to questions regarding participants' main motives were analyzed. For all statistical analysis, an alpha level threshold of 0.05 was used to determine significance. To establish if a difference in rank existed across the motives, a two‐tailed Friedman's two‐way analysis of variance by Ranks was performed with two‐tailed Bonferroni adjusted Wilcoxon Signed Rank tests (Power = 0.80; *α* = 0.05; |*p* | = 0.03; *n* = 767). To investigate the effect of lockdown on a habitually trained CrossFit™ population the responses to questions comparing levels of exercise, motivation, stress, and relaxation, between two scenarios: normal versus lockdown; and first lockdown and second lockdown; were analyzed. To determine differences within each of the groups (defined above), a two‐tailed Mann–Whitney *U* test was performed if there were two subgroups (gender), and a two‐tailed Kruskal–Wallis *H* test with Bonferroni adjusted Mann–Whitney *U* tests employed if there were more than two subgroups (ethnicity, age, and BMI). If the differences were significant between subgroups, effect sizes (ES) were calculated and post hoc power analyses were performed to determine the achieved power of each post hoc test. ES were classified as negligible (0.00–0.001), weak (0.01–0.04), moderate (0.04–0.16), relatively strong (0.16–0.36), strong (0.36–0.64), and very strong (0.64–1.00).

A full STROBE cross‐sectional checklist available at from the corresponding author, detailing further details of the methodological design can be seen in Appendix 2 available at request from corresponding author.

## 3 RESULTS

### 3.1 Participant characteristics

A total of 757 participants (height: 1.71 ± 0.10 m; weight: 76.4 ± 16.1 kg; BMI: 26.1 ± 4.7 kg/m^2^) completed the online survey in full. There was a slight skew in gender within the respondents with 59.2% identifying as female compared with 40.8% as male (Table 1). Most participants, 73.2%, were aged between 25 and 44 years, with 36.7% aged 25–34 years, and 36.5% aged 35–44 years (Table 1). Furthermore, a heavy ethical bias in responders was observed with 93.9% identifying as White compared with 1.7% Asian, 3.3% Mixed, and 1% Other (Table 1).

**Table 1:** Participant characteristics.

Category	*n*	%
Gender		
Male	309	40.8
Female	448	59.2
Age (years)		
18–24	44	5.8
25–34	278	36.7
35–44	276	36.5
45–54	131	17.3
55–64	21	2.8
65+	7	0.9
Ethnicity		
White	711	93.9
Asian	13	1.7
Mixed	25	3.3
Other	8	1.1
Body mass ranking		
Underweight	7	0.9
Normal	338	45.1
Overweight	306	40.8
Obese 1	68	9.1
Obese 2	16	2.1
Obese 3	15	2.0

Abbreviations: *n*, sample size; %, percentage.

### 3.2 CrossFit™ participation and training experience

Participants reported their current training from <1 year experience to 5+ years. Almost half of the participants reported training for over 5 years and only 8% had less than 1 year's experience (see Table 2). In terms of specific CrossFit™ training, participants reported less years' experience with only 21% having 5+ years in a CrossFit™ gym. However, 94% of all respondents reported attending at least three CrossFit™ sessions per week with 13% attending six sessions and 11% attending every day (see Table 2).

**Table 2:** Training experience and participation.

Category	*n*	%
Training age		
<1 year experience	61	8
1–2 years experience	91	12
2–3 years experience	105	14
3–4 years experience	91	12
4–5 years experience	61	8
5 years +	348	46
Minutes of exercise per week		
< 60 min	8	1
60–120 min	45	6
120–180 min	121	16
180–240 min	174	23
240+ mins	409	54
CrossFit™ training age		
< 1 year	167	22
1–2 years	151	20
2–3 years	136	18
3–4 years	91	12
4–5 years	53	7
5+ years	159	21
Number of CrossFit™ sessions per week		
1 p/w	8	1
2 p/w	37	5
3 p/w	167	22
4 p/w	167	22
5 p/w	197	26
6 p/w	98	13
7 + p/w	83	11

Abbreviations: *n*, sample size; %, percentage.

### 3.3 Motives for CrossFit™ participation

**Figure 1:**
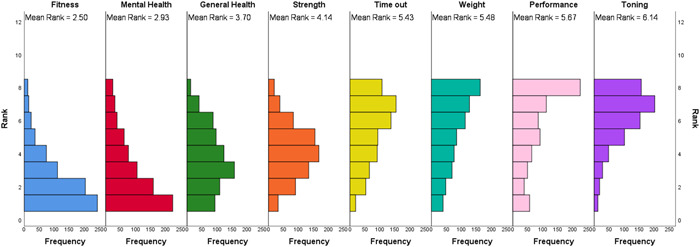
Mean rank and distribution of the ranking of motives for attending the box of CrossFit™ gym members in the United Kingdom (1 = most important, 8 = least important).

Participants were asked to rank their main motives for attending the box in order from 1 = most important, 8 = least important (Figure 1). Results showed that CrossFit™ participants ranked fitness (2.55 ± 1.72) as their main motive for attending the box, however, mental health (2.92 ± 2.02) was ranked a close second, with general health benefits (3.71 ± 1.83) being ranked the third most important motive, strength (4.14 ± 1.63) ranked fourth, time out (5.43 ± 1.95) fifth, weight management/loss (5.46 ± 2.15) sixth, athletic performance (5.66 ± 2.28) seventh, and finally the motive which CrossFit™ participants ranked the least important was toning (6.13 ± 1.66).

There was a significant difference found in the distribution of ranks given to the motives for CrossFit Participation (*χ* ^2^(7) = 1571.5, *p* < 0.001; Figure 1). Post hoc analysis with a Bonferroni correction applied identified significant differences (*Ζ* = −20.77 to −3.91, *p* < 0.003; Figure 1) in the ranking of fitness, mental health, general health benefits, strength, and toning with all other motives, the strength of the ES of these differences were moderate to very strong (0.14–0.75). No significant differences were found (*Z* = −2.09 to −0.605, *p* > 0.05; Figure 1) in the rank between the following motives: time out; weight management/loss, and athletic performance, with the ES classified as weak (0.02–0.08).

### 3.4 Exercise behaviors during the second lockdown

#### 3.4.1 Effect of a second lockdown on exercise levels, motivation to train, and feelings of stress

**Figure 2:**
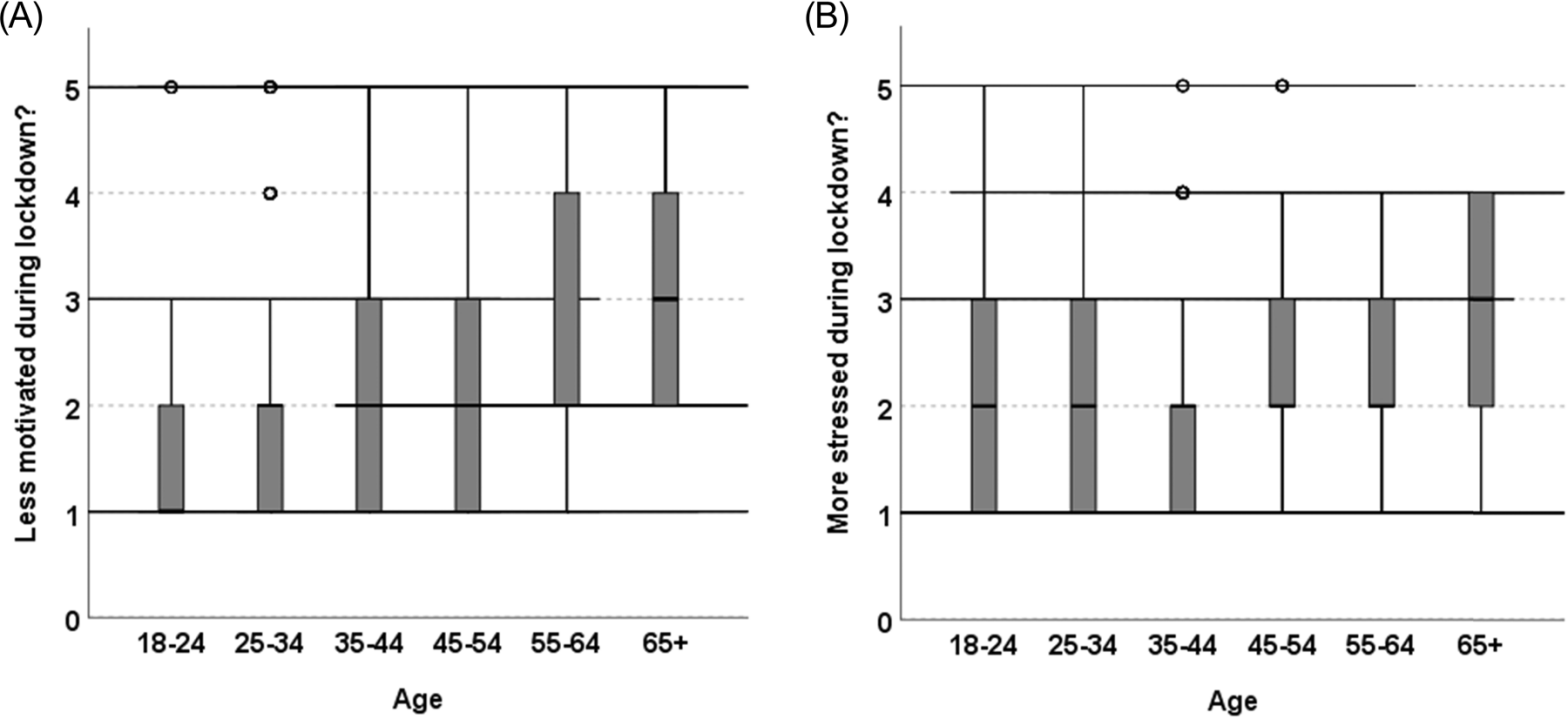
Median levels of agreement reported between age groups for: (A) were your motivation levels to train less during lockdown compared with normal? (B) did you feel more stressed during lockdown compared with normal?

Respondents reported that during lockdown they exercised less and had less motivation to train at home compared with before lockdown (Table 3). They also reported feeling more stressed during lockdown compared with pre‐lockdown (Table 3). These responses were common across genders, ethnicity, and BMI groups with no significant differences found in the strength of agreement between the subgroups. Significant differences were found however, across the age subgroups regarding a lower motivation to train at home (*χ* ^2^(4) = 35.45, *p* < 0.001; Figure 2A) and feeling more stressed (*χ* ^2^(4) = 16.84, *p* = 0.002; Figure 2B) compared with pre‐lockdown. No significant differences were found regarding the strength of agreement on exercising less due to lockdown across the age subgroups.

**Table 3:** Median and interquartile range (IQR) for the effect of lockdown on pre‐lockdown exercise levels, motivation to train at home, and feelings of stress for all participants and across gender, ethnicity, age, and BMI demographics.

	Less exercise?		Less motivation to train?		Feeling more stressed?	
Enrollment	Median	IQR	Median	IQR	Median	IQR
All	2.0	1.0–4.0	2.0	1.0–3.0	2.0	1.0–3.0
Gender						
Females	2.0	1.0–4.0	2.0	1.0–3.0	2.0	1.0–2.75
Males	2.0	1.0–4.0	2.0	1.0–2.0	2.0	1.0–3.0
Ethnicity						
White	2.0	1.0–4.0	2.0	1.0–3.0	2.0	1.0–3.0
Asian	2.0	1.0–2.0	2.0	1.0–3.0	2.0	1.0–2.0
Mixed	2.0	1.25–4.0	1.0	1.0–2.5	2.0	1.5–3.5
Other	3.5	1.0–4.0	1.5	1.5–3.5	1.5	1.0–2.75
Age						
18–24	2.0	1.0–3.0	1.0	1.0–2.0	2.0	1.0–3.0
25–34	2.0	1.0–3.25	2.0	1.0–2.0	2.0	1.0–3.0
35–44	2.0	1.0–4.0	2.0	1.0–3.0	2.0	1.0–2.0
45–54	2.0	1.0–4.0	2.0	1.0–3.0	2.0	2.0–3.0
55–64	2.0	2.0–4.0	2.0	2.0–4.0	2.0	2.0–3.0
65 +	4.0	2.0–5.0	3.0	2.0–4.0	3.0	2.0–4.0
BMI						
Underweight	3.0	1.0–4.0	2.0	2.0–2.0	2.0	1.0–3.0
Normal	2.0	1.0–4.0	2.0	1.0–3.0	2.0	1.0–3.0
Overweight	2.0	1.0–4.0	2.0	1.0–2.0	2.0	1.0–3.0
Obese I	2.0	1.0–3.0	1.0	1.0–2.0	2.0	1.0–3.0
Obese II	2.0	1.0–3.5	2.0	1.25–2.75	2.0	1.0–3.0
Obese III	1.0	1.0–2.0	2.0	1.0–3.0	2.0	1.0–2.0

*Note*: Scale = 1.0, strongly agree; 2.0, agree; 3.0, neither agree nor disagree; 4.0, disagree; 5.0, strongly disagree.

Abbreviation: BMI, body mass index.

Post hoc tests investigating the strength of agreement on the lack of motivation to train at home during lockdown highlighted significant differences between the following subgroups: 18–24 versus 35–24 (*U* = 4157, *p* = 0.006; ES: 0.13; Power = 0.12), 18–24 versus 45–54 (*U* = 1796.5, *p* = 0.002; ES: 0.14; Power = 0.12), 18–24 versus 55–64 (*U* = 237 *p* = 0.009; ES: 0.12; Power = 0.07); and 25–34 versus 45–54 (*U* = 4157, *p* = 0.006; ES: 0.13; Power = 0.25). No further significant differences between age subgroups were identified. Despite the low reliability of these findings due to the large differences in subgroup population, there is some evidence that the younger age groups (18–24 and 25–34) more strongly agreed that their motivation levels to train at home during lockdown were less compared with pre‐lockdown, than the older age subgroups. Although the difference in median between the over 65 age group (which indicated they neither agreed nor disagreed that their motivation levels to train at home were less during lockdown compared with pre‐lockdown) and the other age groups was visibly different, no significant differences were observed likely due to the small population (*n* = 7) and large variation in responses.

Similarly, post hoc tests investigating the strength of agreement regarding feeling more stressed during lockdown highlighted significant differences between the 25–34 and 45–54 age subgroups (*U* = 37184, *p* = 0.004; ES = 0.13; Power = 0.22). Despite the low reliability of these findings due to the large differences in subgroup population, there is some evidence that the younger age group (25–34) felt more stressed during lockdown than the older age group (45–54). Again, despite the median indicating the over 65 age group neither agreed nor disagreed that they felt more stressed during lockdown compared with pre‐lockdown, no significant differences were observed.

#### 3.4.2 Effect of second lockdown on first lockdown exercise levels, motivation to train, and feelings of stress

**Figure 3:**
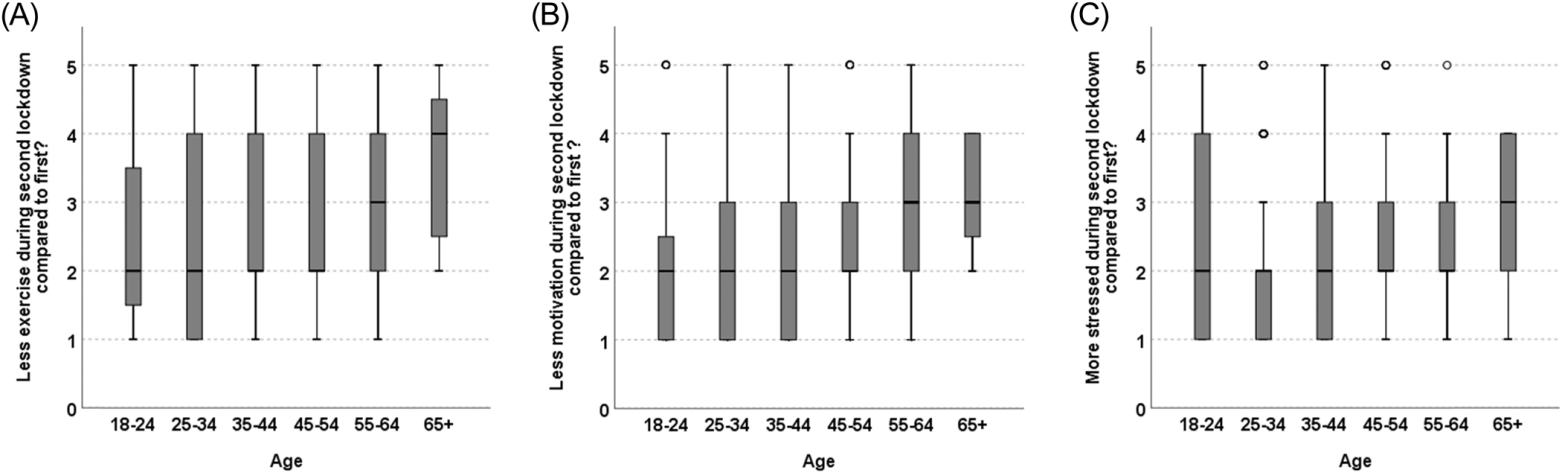
Median levels of agreement reported between age groups for: (A) were your exercise levels less during the second lockdown compared with the first? (B) were your motivation levels to train less during the second lockdown compared with the first? (C) did you feel more stressed during the second lockdown compared to first?

Respondents demonstrated a level of agreement that during the second lockdown they exercised less and had less motivation to train at home compared with the first lockdown (Table 4). They also agreed that they felt more stressed during the second lockdown compared with the first (Table 4). These findings were again common across genders, ethnicity, and BMI groups with no significant differences found in the strength of agreement between the subgroups. Significant differences were found across age subgroups regarding the strength of agreement relating to exercising less (*χ* ^2^(4) = 13.77, *p* = 0.008; Figure 3A), the motivation to train at home being less (*χ* ^2^(4) = 20.03, *p* < 0.001; Figure 3B) and the feeling of being more stressed (*χ* ^2^(4) = 12.90, *p* = 0.01; Figure 3C) during the second lockdown compared with the first lockdown.

**Table 4:** Median and interquartile range (IQR) for the effect of the second lockdown on first lockdown exercise levels, motivation to train at home, and feelings of stress for all participants and across gender, ethnicity, age, and BMI demographics.

	Less exercise?		Less motivation to train?		Feeling more stressed?	
Enrollment	Median	IQR	Median	IQR	Median	IQR
All	2.0	1.0–4.0	2.0	1.0–3.0	2.0	1.0–3.0
Gender						
Females	2.0	1.75–4.0	2.0	1.0–3.0	2.0	1.0–3.0
Males	2.0	1.0–4.0	2.0	1.0–3.0	2.0	1.0–3.0
Ethnicity						
White	2.0	1.0–4.0	2.0	1.0–3.0	2.0	1.0–3.0
Asian	2.0	1.0–3.5	1.0	1.0–2.5	1.0	1.0–2.5
Mixed	2.0	1.0–3.75	1.0	1.0–3.0	2.0	1.0–2.5
Other	2.5	1.0–4.0	2.0	1.0–3.0	2.0	1.0–3.0
Age						
18–24	2.0	1.25–3.75	2.0	1.0–2.75	2.0	1.0–4.0
25–34	2.0	1.0–4.0	2.0	1.0–3.0	2.0	1.0–2.0
35–44	2.0	2.0–4.0	2.0	1.0–3.0	2.0	1.0–3.0
45–54	2.0	2.0–4.0	2.0	2.0–3.25	2.0	2.0–3.0
55–64	3.0	2.0–4.0	3.0	2.0–4.0	2.0	1.5–3.0
65 +	4.0	2.0–5.0	3.0	2.0–4.0	3.0	2.0–4.0
BMI						
Underweight	2.0	1.0–2.0	2.0	1.0–2.0	2.0	1.0–4.0
Normal	2.0	2.0–4.0	2.0	1.0–3.0	2.0	1.0–3.0
Overweight	2.0	1.75–4.0	2.0	1.0–3.0	2.0	1.0– 3.0
Obese I	2.0	1.0–4.0	1.0	1.0–3.0	2.0	1.0–2.75
Obese II	2.0	1.0–4.0	2.0	2.0–4.0	2.5	1.0–3.0
Obese III	3.0	1.0–3.0	3.0	1.0–3.0	2.0	1.0–3.0

*Note*: Scale = 1.0, strongly agree; 2.0, agree; 3.0, neither agree nor disagree; 4.0, disagree; 5.0, strongly disagree.

Abbreviation: BMI, body mass index.

Despite there being a significant effect of age on all three factors globally, post hoc tests were unable to reveal significant differences between any of the subgroups when the alpha value had been adjusted for multiple comparisons using Bonferroni's correction. This is likely due to the low power of each statistical test due to the large differences in each subgroup population size. Despite differences in the median between age groups within each factor, especially in the older age groups (55–64 and 65 + ), observation of the interquartile range within each group (Figure 3) highlights a large variation in responses.

## 4 DISCUSSION

The purpose of this article was to evaluate the effects of the National UK lockdowns on the behavior, motive, and general health and well‐being of habitually trained CrossFit™ participants. Specifically, emotional stress and exercise behaviors were investigated to determine how this group of individuals responded to a second lockdown period. Despite a recent publication^18^ indicating that exercise habits remained unchanged during the first UK National lockdown, the current study found that habitually trained CrossFit™ participants were more likely to self‐report greater levels of emotional stress and decreased motivation to exercise during the second UK national lockdown. In contrast to previous studies which have prominently investigated lockdowns in isolation and/or focused on untrained individuals^8^, ^16^ the current study suggests that the second lockdown was detrimental to both the mental and physical health of those who meet or exceed typical exercise guidelines and thus regard it as key to their lifestyle.

### 4.1 Stress

Periods of lockdown, such as those experienced in the United Kingdom have previously been associated with increased stress, anxiety, depression, anger, and confusion.^19^ Stress is reportedly an outcome of economic and financial uncertainty, and a disruption to, or change to normal routine.^22^, ^23^, ^24^, ^25^ The removal of favored activities, or inability to visit “favorite places” has also been associated with increased feelings of stress in habitual exercisers.^19^

In the current study, stress was reported as greater in the second lockdown compared with the first, specifically participants were generally less stressed and experienced more positive mental and physical well‐being during the first lockdown compared with the second. Interestingly the youngest age group (18–24 years) were more likely to report increased stress during the second UK national lockdown, which is somewhat concerning.

A possible explanation for the increase in stress could relate to the reasons participants gave for their consistent participation in CrossFit™. Several participants in the current study ranked mental health as one of the highest reasons for participation, indicating that managing mental health through consistent exercise is an essential for many and as such, restricting habitual exercise patterns could be synonymous with increased feelings of stress. This relationship is also highlighted in previous literature^24^ and has also been associated more strongly with younger adults. Namely as the disruption to their exercise routine results in a more significant change to their everyday life compared with their older counterparts, due to the reduction in social interaction, worry relating to the pandemic and a loss of local support network.^23^, ^24^, ^25^ Furthermore, reductions in habitual exercise are often associated with feelings of guilt thus it may be evident that participants were experiencing emotional distress/guilt associated with a reduction to their normal exercise habits.^19^ No further differences were noted in genders, despite contemporary literature indicating that younger females are at greater risk of pandemic induced anxiety and depression.^2^, ^26^

Typically, the CrossFit™ program is thought to enhance aspects of psychological health through creating a social support network.^21^ High levels of motivation to exercise are considered to occur in tandem with social support and a sense of belongingness.^27^, ^28^, ^29^ The results of the current study indicate that even the localized online support of a CrossFit™ community could not offset the increased feelings of stress in habitually trained individuals during the second lockdown period, when access to the gym was restricted. Previous research^18^ would suggest that habitually trained CrossFit™ participants were more protected in their mental health during lockdown periods, particularly when compared with other forms of exercise.^20^ The current study confirms that this is not the case when multiple lockdowns occur suggesting that cessation from community‐focused activity, and prolonged lockdown periods are a risk to mental health.

An inverse relationship between positive healthy behaviors and emotional distress has been identified.^1^, ^19^, ^23^, ^24^, ^25^ This is supported by results of the current study. Thus, it is evident that government‐imposed lockdowns can have a significant impact on the psychological welfare of its constituents when normal healthy behaviors and routines are disrupted.^29^

### 4.2 Exercise behavior

Studies have reported numerous behavioral changes in UK residents following imposed restrictions.^6^, ^30^ These include changes to eating habits, alcohol consumption,^30^, ^31^ and observed exercise habits,^30^ though no studies to date have investigated the impact of a lockdown in habitually trained athletes, let alone across different age groups. The current study indicates that the younger respondents (18–24 and 25–34 years) were less motivated to exercise, reporting the greatest reduction in motivation during the second lockdown compared with their older counterparts. Recently studies^32^ have hypothesized that older age groups maybe more likely to continue exercising, due to perceptions of COVID‐19 infection risk however no conclusions in the literature have been found as to why younger participants were impacted more readily by lockdown periods and potentially points to the importance of a social network for younger generations.

Other reasons for changing exercise behavior may include access to equipment and a suitable training environment. During lockdown, individuals were required to exercise at home in either indoor living space or outdoors. Many habitual exercisers were able to mitigate feelings of stress during initial lockdown periods, by modifying their habitual exercise practice when favored facilities were inaccessible.^19^ However, it is documented that habitual exercise behaviors change during colder months in tandem with exercise motivation, despite the increased risk of chronic disease and illness during this period.^33^ It seems reasonable to suggest that lockdown periods during the colder, darker months may have impacted individual desire to exercise, thereby decreasing overall participation rates.

The outcome of this, and the associated loss of exercise behaviors is likely to translate to health and disease, as evidenced by reports noting increases in obesity/BMI levels during the pandemic.^12^, ^14^ decreased motivation to eat healthy food,^31^ decreased motivation to exercise,^7^ decreased mental health,^1^, ^2^, ^3^ and increased alcohol consumption.^8^

Previous research into CrossFit™ participation has found associated increases in mental and physical resilience,^27^ as well as a reduction in perceived severity of COVID‐19 symptoms.^18^ Yet even in habitually trained CrossFit™ participants, frequent government‐imposed restrictions can have harmful effects on healthy behaviors which would otherwise lead to enhanced physical and mental well‐being. The findings in the current study support the argument that fitness facilities should operate as an essential business, with reduced capacity, to support the ongoing physical and mental health of members during lockdown periods and reduce the mirage of associated health decline related to such restrictions.

### 4.3 Strengths & limitations

While the current study provides a comprehensive analysis of exercise behaviors during the second government‐imposed lockdown, it does have several limitations. First, the survey did not consider additional variables that may have caused stress and or decreases in motivation during the lockdown periods such as loss of work/income, fear of illness, and so on. Although this may have influenced participants responses to questions related to stress, motivation, and behaviors, the nature of field work such as this means to control all external factors would be beyond the scope of the data collection methods. As the aim of the study was to compare the two UK lockdowns a number of these confounding variables would be present across both periods. The survey also specifically asked about stress, motivation, behavior, and so on, related to a lack of physical activity, which was highlighted at the start of the questions. Second, participants were recruited via social media platforms to complete an online questionnaire. Therefore, the sample may be biased toward those who have previously used similar technologies. In addition, a further bias may exist within the findings due to the unequal size of the groups when participants were grouped based on age, ethnicity, BMI or training experience and participation, which may limit the power of the statistical approach adopted. However, the strategy for recruitment and participant was the most feasible at the time of completion. Furthermore, the recruitment strategy also relied on self‐selection to participate and therefore there may be some evidence of bias to those interested in the effect that national UK lockdown had on CrossFit™ participation. Nonetheless, this was a particular area of interest for researchers, therefore it did not seem appropriate to recruit beyond the CrossFit™ community.

## 5 PERSPECTIVE

Overall, the purpose of this study was to investigate the effects of consecutive lockdown periods in a habitually trained population. Although previous research has highlighted no impact on habitual exercisers, results from the current study found that the behavior, motives, and general health of members were impacted by the second lockdown, with a more significant impact on the younger age groups. These individuals (18–24 year age group) reported lower motivation to exercise and increased stress levels during the second lockdown, indicating their potential risk of decreased physical and psychological well‐being. This was attributed to the decrease in social interaction, which is vital to younger age groups, especially when their social network revolves around their physical activity habits. It is argued that these factors need to be addressed in planning for future national lockdowns, and consideration should be given to recognizing fitness facilities as essential practices within healthcare during imposed restrictions or prolonged periods of imposed closure especially given the link between physical activity and COVID‐19 severity.^18^

## AUTHOR CONTRIBUTIONS

**Athalie Redwood‐Brown**: Conceptualization; investigation; methodology; project administration; writing — original draft; writing — review & editing. **Jennifer Wilson**: Conceptualization; methodology; writing — original draft; writing — review & editing. **Paul Felton**: Formal analysis; writing — review & editing.

## CONFLICT OF INTEREST STATEMENT

The authors declare no conflict of interest.

## TRANSPARENCY STATEMENT

The lead author Athalie Redwood Brown affirms that this manuscript is an honest, accurate, and transparent account of the study being reported; that no important aspects of the study have been omitted; and that any discrepancies from the study as planned (and, if relevant, registered) have been explained.

## ETHICS STATEMENT

The study protocol was reviewed and approved by Nottingham Trent Universities Noninvasive Ethics Committee. Before completing the survey, participants were given details on the purpose and aims of the study and were requested to give their informed consent. All authors have read and approved the final version of the manuscript and had full access to all the data in this study and take complete responsibility for the integrity of the data and the accuracy of the data analysis.

## Data Availability

The data that support the findings of this study are available on request from the corresponding author. The data are not publicly available due to privacy or ethical restrictions.
